# Recombinant Thrombomodulin Suppresses Histone-Induced Neutrophil Extracellular Trap Formation

**DOI:** 10.3389/fimmu.2019.02535

**Published:** 2019-10-29

**Authors:** Binita Shrestha, Takashi Ito, Midori Kakuuchi, Takaaki Totoki, Tomoka Nagasato, Mika Yamamoto, Ikuro Maruyama

**Affiliations:** ^1^Department of Systems Biology in Thromboregulation, Kagoshima University Graduate School of Medical and Dental Sciences, Kagoshima, Japan; ^2^Department of Medicinal Chemistry, Rogel Cancer Center, College of Pharmacy, University of Michigan, Ann Arbor, MI, United States; ^3^Department of Emergency and Intensive Care Medicine, Kagoshima University Graduate School of Medical and Dental Sciences, Kagoshima, Japan

**Keywords:** histone, NETs, thrombomodulin, thrombosis, neutrophil

## Abstract

Histones, the major protein components of chromatin, are released into the extracellular space during sepsis, trauma, and ischemia-reperfusion injury, and subsequently mediate organ failure. Extracellular histones can promote endothelial damage and platelet aggregation, which can be suppressed by administration of recombinant thrombomodulin (rTM). The present study aimed to clarify whether histones can activate neutrophils to induce NET formation and whether rTM can prevent histone-induced NET formation. NET formation was analyzed *in vitro* by stimulating human neutrophils with histones in the absence or presence of rTM. NET formation was further analyzed *in vivo* by intravenous infusion of histones into rats with or without rTM. Histones induced NET release in a dose-dependent manner *in vitro* and NET release was induced as early as 1 h after stimulation. Histone-induced NET release was independent of NADPH oxidase. rTM suppressed histone-induced NET release *in vitro* as well as *in vivo*. The suppression might be mediated by rTM binding to histones, as suggested by analysis using a quartz crystal microbalance system. The present findings suggest that histones can activate neutrophils to form NETs and that rTM can inhibit histone-induced NET formation.

## Introduction

Neutrophils are the first line of innate immune defense against infectious agents. In addition to their ability to eliminate pathogens by phagocytosis and/or degranulation, neutrophils can bind and kill a wide range of microorganisms by producing neutrophil extracellular traps (NETs) (1–3). NETs are formed through release of antimicrobial proteins anchored to a chromatin network by activated neutrophils, and subsequently ensnare invading bacteria within capillary beds to prevent microbial dissemination (4, 5). Although NET release is an important antimicrobial mechanism, there is growing evidence that NET formation contributes to the pathogenesis of several clinical conditions, including thrombosis, sepsis, and acute lung injury (6–12).

NETs are formed in response to various stimuli. Phorbol myristate acetate (PMA) is a non-physiological stimulus commonly used for *in vitro* studies on NET release. Bacteria, fungi, microbial products such as lipopolysaccharide (LPS), cytokines, and damage-associated molecular patterns (DAMPs) also induce NET release (4, 13, 14). Histones, the major protein components of chromatin, were identified as a new class of DAMPs that cause organ injury through TLRs and directly induce epithelial and endothelial cell death when released into the extracellular space (15–18). Histones are released into the extracellular space during sepsis, trauma, and ischemia-reperfusion injury (18–21). Although some reports suggested that extracellular histones activate neutrophils to induce NET release (22), the cellular and molecular basis of histone-induced organ injury is not yet clear.

Thrombomodulin is an anticoagulant protein that is mainly expressed on the surface of endothelial cells (23). In addition to its anticoagulant activity, thrombomodulin has anti-inflammatory and cytoprotective effects (24, 25). Recombinant thrombomodulin (rTM) has been used for treatment of patients with disseminated intravascular coagulation (DIC) in Japan (24). Recent studies have suggested that rTM protects neutrophils against LPS-induced NET release (26) and protects mice against NET accumulation after intestinal ischemia-reperfusion (27). In the present study, we analyzed whether histones can induce NET release and whether rTM can suppress histone-induced NET release.

## Materials and Methods

### Neutrophil Isolation From Whole Human Blood

All experiments involving human blood were carried out in accordance with the provisions of the Declaration of Helsinki and were approved by the Ethics Committee of Kagoshima University. Written informed consent for participation in the study was obtained from all individuals. For each experiment, primary human neutrophils were freshly isolated from EDTA-anticoagulated venous blood of healthy volunteers using an EasySep Direct Human Neutrophil Isolation Kit (StemCell Technologies, Vancouver, BC, Canada) according to the manufacturer's instructions.

### Neutrophil Activation and NET Detection by Immunolabeling

Neutrophils were seeded in poly-d-lysine-coated 4-well-culture slides at 2.5 × 10^5^ cells/well in 500 μl of RPMI medium (Nacalai tesque Inc., Kyoto, Japan) containing 2% human serum albumin (Lee Biosolutions Inc., Maryland Heights, MO) and incubated in a CO_2_ incubator at 37°C for 1 h. After the incubation, the supernatant was aspirated and 500 μl of Opti-MEM medium (Gibco, NY) without human serum albumin was added. The cells were incubated for 30 min with or without inhibitors of NET formation, rTM or rTM type 2 (Asahi Kasei Pharma Corporation, Tokyo, Japan), and then left unstimulated or stimulated with PMA or combinations of histone H3 and histone H4 for 0, 1, 2, or 4 h. Subsequently, the cells were fixed with 2% paraformaldehyde (PFA), permeabilized with 0.5% Triton X-100 at room temperature for 1 min, blocked with 1% BSA in phosphate-buffered saline (PBS) containing 0.1% Triton X-100 at room temperature for 1 h, and incubated overnight at 4°C with primary antibodies: rabbit anti-histone H3 (citrulline 2 + 8 + 17) polyclonal antibody (1:250 dilution; Abcam, Cambridge, UK), and rabbit anti-neutrophil elastase polyclonal antibodies (1:200 dilution; Calbiochem, La Jolla, CA). The bound primary antibodies were detected by incubation with secondary antibodies coupled to Alexa Fluor 488 or Alexa Fluor 596 (Invitrogen, Eugene, OR) for 1 h at room temperature. For DNA detection, nuclei were stained with DAPI. Cells were analyzed with an LSM700 confocal laser microscope (Carl Zeiss, Oberkochen, Germany).

### Quantification of DNA Release From Activated Neutrophils

Freshly isolated neutrophils were immediately seeded in 96-well black plates (2 × 10^5^ cells/well) in the presence of 5 μM Sytox Green (Life Technologies, Eugene, OR), a non-cell-permeable DNA binding dye, with or without different inhibitors. The cells were then stimulated with PMA or combinations of histone H3 and histone H4 and incubated at 37°C under 5% CO_2_ in the dark for 4 h. Fluorescence was quantified with excitation at 485 nm and emission at 535 nm using an Infinite M200 (Tecan, Austria GmbH).

### Neutrophil Viability Assay

Neutrophils in poly-d-lysine-coated 4-well-culture slides were incubated in the presence or absence of histone H3/H4 and rTM. Live cells were labeled with 1 μM of calcein acetoxymethyl ester (Dojindo Laboratories, Kumamoto, Japan) and dead cells were labeled with 2 μM of propidium iodide (Dojindo Laboratories). Cells were analyzed with fluorescence microscopy BZ-X700 (Keyence, Osaka, Japan) once every minute for the assessment of dynamic change of viability.

### *In vitro* Platelet Aggregation Assays

Citrated blood samples were obtained from healthy volunteers who had not taken any medications that might affect platelet function or coagulation in the preceding 2 weeks. Washed platelets were resuspended in Tyrode-HEPES buffer (pH 7.35), incubated with rTM or rTM2 at various concentrations (0.5–10 μM), and stimulated with 1 μM histone H4. Platelet aggregation was optically monitored using a light transmission aggregometer (MCM Hema Tracer 313M; SSR Engineering, Tokyo, Japan).

### Coomassie Brilliant Blue Staining

rTM and rTM2 were incubated with various amounts of chondroitinase ABC (1–1,000 mU) for defined time intervals (5–60 min). Removal of chondroitin sulfate from rTM2 was confirmed by sodium dodecyl sulfate -polyacrylamide gel electrophoresis followed by Coomassie brilliant blue staining (Wako, Japan).

### Binding Assays With a Quartz Crystal Microbalance (QCM) Twin Sensor System

The interactions between histones and rTM were assessed with a NAPiCOS Auto QCM Twin Sensor System (Nihon Dempa Kogyo Co., Tokyo, Japan) as previously described (28) with slight modifications. Briefly, one channel on a sensor chip was coated with rTM (20 μM), and a second channel on the same sensor chip was coated with rTM2 (20 μM). The sensor chip was washed three times with PBS (pH 7.4), placed in the NAPiCOS Auto QCM instrument, and perfused with histone H3 (50 μM) or histone H4 (50 μM). In some experiments, both channels on a sensor chip were coated with rTM (20 μM) or rTM2 (20 μM) and one channel was incubated with 100 mU of chondroitinase ABC (Sigma-Aldrich) and the other channel was incubated with buffer. The sensor chip was washed three times with PBS (pH 7.4), placed in the NAPiCOS Auto QCM instrument, and perfused with histone H3 (50 μM) or histone H4 (50 μM). The interactions between the molecules were recognized as changes in frequency of a quartz crystal resonator. All experiments were carried out at 25°C with a flow rate of 5 μl/min.

### Animal Experiments

Central intravenous catheters were placed in 9-week-old male Sprague-Dawley rats (Charles River Laboratories, Atsugi, Japan). At 10 weeks of age, these rats were divided into four groups. Group 1 was given a continuous intravenous infusion of saline for 240 min and a bolus injection of saline at 120 min. Group 2 was given a continuous intravenous infusion of saline for 240 min and a bolus injection of 1 mg/kg rTM at 120 min. Group 3 was given a continuous intravenous infusion of 0.5 mg/kg/min calf thymus histones (Sigma-Aldrich, St Louis, MO) for 240 min and a bolus injection of saline at 120 min. Group 4 was given a continuous intravenous infusion of 0.5 mg/kg/min calf thymus histones for 240 min and a bolus injection of 1 mg/kg rTM at 120 min. Blood samples were collected from central vein catheter every 60 min, and tissue samples were collected from euthanized rats soon after the end of histone infusion. All animal experiments were performed in accordance with the guidelines of Shin Nippon Biomedical Laboratories and Kagoshima University, Kagoshima, Japan.

### Immunohistochemical Staining of Rat Tissue Sections

For immunohistochemistry, paraffin sections were heated in a microwave oven for 20 min, dewaxed in xylene and rehydrated through a graded series of ethanol solutions. Endogenous peroxidase activity was blocked by incubation with 0.3% hydrogen peroxide in absolute methanol for 15 min at room temperature. Antigen epitopes were heat-retrieved in Antigen Unmasking Solution (Vector Laboratories Inc., Burlingame, CA), and the sections were incubated overnight at 4°C with primary antibodies: rabbit anti-histone H3 (citrulline 2 + 8 + 17) polyclonal antibody (1:250 dilution, Abcam) and goat polyclonal anti-myeloperoxidase (MPO) heavy chain (1:50 dilution; Santa Cruz Biotechnology Inc., Santa Cruz, CA). The primary antibodies were diluted in PBS containing 0.01% Tween-20 and 1% BSA. Subsequently, the sections were incubated with secondary antibodies using Histofine Simple Stain Mouse MAX-PO (Rabbit) or Histofine Simple Stain Mouse MAX-PO (Goat) (Nichirei, Tokyo, Japan) for 1 h at room temperature. Peroxidase activity was visualized with 3′3-diaminobenzidine (Dako North America Inc., Carpenteria, CA), and the sections were lightly counter-stained with Lillie-Meyer's hematoxylin (Wako, Osaka, Japan). Images were obtained using an Axiophot microscope equipped with an AxioCam MRc5 camera and AxioVision Release 4.6 software (Carl Zeiss).

### Immunofluorescence Staining of Rat Tissue Sections

For immunofluorescence staining, paraffin sections were heated in a microwave oven for 20 min, dewaxed in xylene, and rehydrated through a graded series of ethanol solutions. Antigen epitopes were heat-retrieved in Antigen Unmasking Solution (Vector Laboratories Inc.), and the sections were blocked with 1% BSA in PBS containing 0.1% Triton X-100 for 1 h at room temperature. The sections were then incubated overnight at 4°C with primary antibodies, rabbit anti-histone H3 (citrulline 2 + 8 + 17) polyclonal antibody (1:250 dilution; Abcam) and goat anti-MPO heavy chain polyclonal antibody (1:50 dilution; Santa Cruz Biotechnology Inc.) followed by incubation with secondary antibodies coupled to Alexa Fluor 488 or Alexa Fluor 596 for 1 h at room temperature. For DNA detection, nuclei were stained with DAPI. The stained sections were analyzed with an LSM700 confocal laser microscope (Carl Zeiss).

### Measurement of Serum Creatinine Levels and Plasma Tumor Necrosis Factor- α Levels

Serum and plasma samples were collected every 60 min during 240 min histone infusion in rats. Serum creatinine levels were determined using BioMajesty JCA-BM6070 (Jeol Ltd., Tokyo, Japan). Plasma tumor necrosis factor-α levels were determined using Rat TNF-alpha ELISA Kit (Bender MedSystems GmbH, Vienna, Austria), according to the manufacturer's instruction.

### Statistical Analysis

Statistical analyses were performed by the Tukey Kramer test, Scheffe's *F*-test, and Bonferroni/Dunn test for all experimental procedures. Values of ^*^p < 0.05 and ^**^p < 0.01 were considered significant.

## Results

### Histone Induces NET Release in Neutrophils

**Figure 1:**
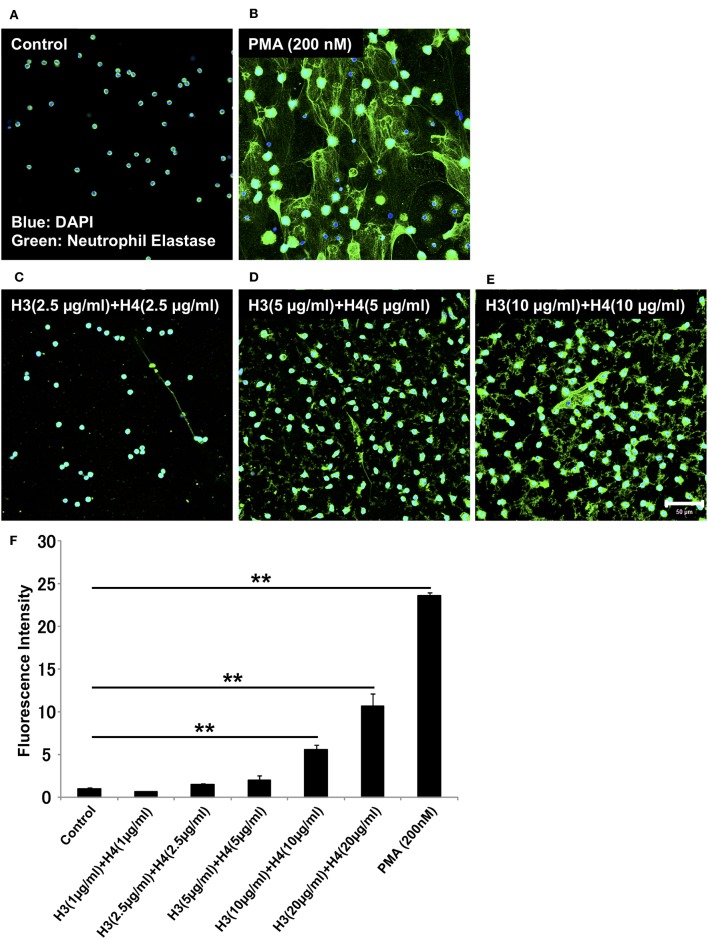
Histones H3 and H4 activate neutrophils to induce NET release. Neutrophils freshly isolated from healthy volunteers were left untreated **(A)**, treated with 200 nM PMA as a positive control **(B)**, or treated with combinations of histones H3 and H4 at the total concentration of 5 μg/ml **(C)**, 10 μg/ml **(D)**, or 20 μg/ml **(E)** for 4 h. The neutrophils were then stained with a primary antibody against neutrophil elastase followed by an Alexa Fluor 488-conjugated secondary antibody. Nuclei were stained with DAPI. The presence of extracellular DNA was quantified by Sytox Green assays **(F)**. Images and graphs are representative of three replicate experiments. Data are means ± SEM (*n* = 4). Scale bar, 50 μm. **p < 0.01.

Histones are detected in plasma from patients with sepsis, and the plasma concentration of histone H3 during sepsis can reach about 15 μg/ml (21). To examine whether histones induce NET release *in vitro*, we stimulated neutrophils isolated from human blood with different doses of histone H3 and histone H4. To allow simple correlations with physiological amounts, we started with H3 1 + H4 1 μg/ml, and gradually increased the dose to H3 20 + H4 20 μg/ml. NET release, as suggested by extracellular DNA co-localized with neutrophil elastase, was not observed in control untreated group (Figure 1A). PMA 200 nM was used as a positive control (Figure 1B). NET-like structures were observed at H3 2.5 + H4 2.5 μg/ml and H3 5 + H4 5 μg/ml, but were prominent at H3 10 + H4 10 μg/ml (Figures 1C–E). Quantification of extracellular DNA levels revealed dose-dependent NET release by histone stimulation (Figure 1F). To further confirm that the released structures were NETs, the cells were immunostained for citrullinated histone H3 (Cit H3), a marker for NET release. As shown in Supplementary Figure 1, the structures were positively stained for Cit H3.

### Histone-Induced NET Release Is Independent of NADPH Oxidase

**Figure 2:**
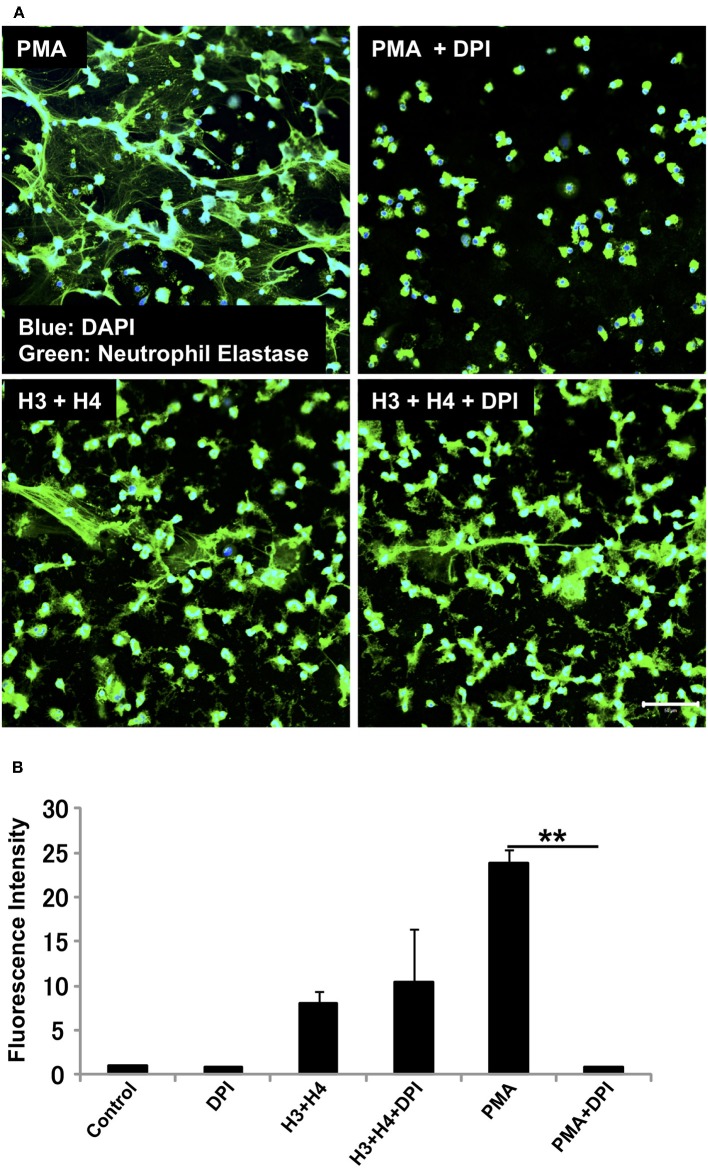
Histone-induced NET release is independent of NADPH oxidase. Neutrophils isolated from healthy volunteers were treated with PMA (200 nM) and histones H3 and H4 (20 μg/ml) in the absence or presence of NADPH oxidase inhibitor DPI **(A)**. The neutrophils were then stained with a primary antibody against neutrophil elastase followed by an Alexa Fluor 488-conjugated secondary antibody. Nuclei were stained with DAPI. The presence of extracellular DNA was quantified by Sytox Green assays **(B)**. Images and graphs are representative of three replicate experiments. Data are means ± SEM (*n* = 4). Scale bar, 50 μm. **p < 0.01.

We investigated whether the mechanism for histone-induced NET release is similar to that for PMA-induced NET release. PMA-induced NET release is NADPH oxidase-dependent, and completely abolished by pre-incubation of neutrophils with NADPH oxidase inhibitor DPI (29). In our experiments, pre-treatment with DPI inhibited PMA-induced NET release, but did not inhibit histone-induced NET release (Figure 2A) indicating that the mechanism for histone-induced NET release was independent of NADPH oxidase. Quantification of total DNA release produced similar results (Figure 2B).

In neutrophils, a metabolic shift toward pentose phosphate pathway fuels NADPH oxidase to induce NET release. Therefore, suppression of glycolysis can suppress NET release (30, 31). Regarding PMA-induced NET release, suppression of glycolysis by 2-deoxyglucose (2-DG) inhibited NET release. In contrast, suppression of glycolysis by 2-DG did not affect histone-induced NET release (Supplementary Figure 2), further indicating that histone-induced NET release was not dependent on NADPH oxidase.

### Histone Induces NET Release in Neutrophils as Early as 1 h After Stimulation

**Figure 3:**
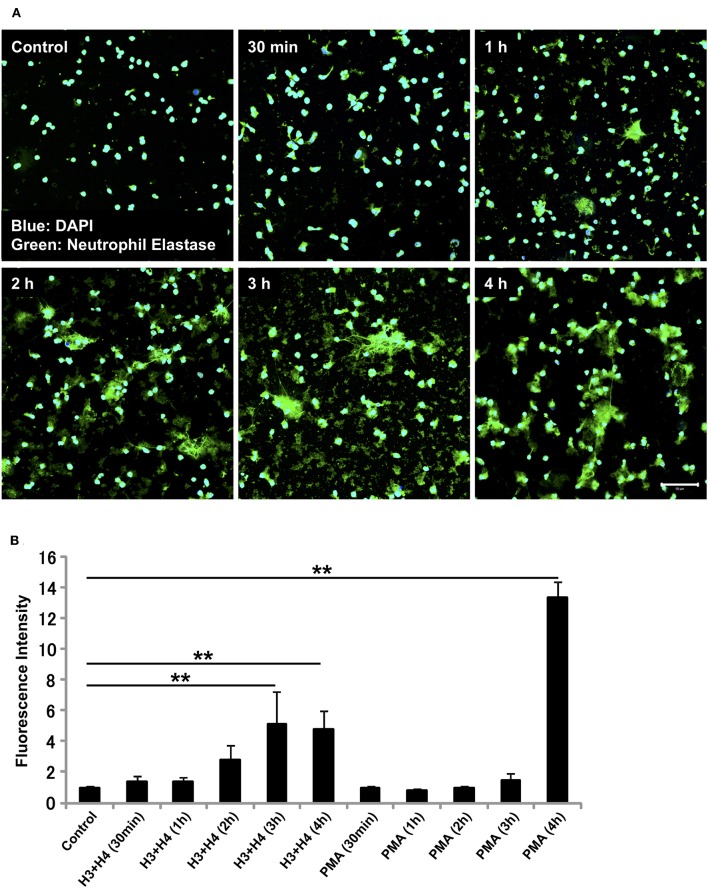
Histone induces NET release as early as 1 h. Neutrophils isolated from healthy volunteers were left untreated or treated with histones H3 and H4 (20 μg/ml) for indicated time intervals **(A)**. The neutrophils were then incubated with a primary antibody against neutrophil elastase followed by an Alexa Fluor 488-conjugated secondary antibody. Nuclei were stained with DAPI. The presence of extracellular DNA was quantified by Sytox Green assays **(B)**. Images and graphs are representative of three replicate experiments. Data are means ± SEM (*n* = 4). Scale bar, 50 μm. **p < 0.01.

The requirement of NADPH oxidase for NET release differs depending on the stimuli received. Similarly, the kinetics of NET release depend on the stimuli involved (13, 32–35). Since histone-induced NET release was not dependent on NADPH oxidase, we evaluated the time-dependent release of NETs by neutrophils after stimulation with histones. In immunostaining, NETs were observed as early as 1 h after stimulation (Figure 3A), and the release of NETs increased in a time-dependent manner (Figure 3B). In contrast, PMA-induced NET release was prominent after 4 h of stimulation (Figure 3B). Immunostaining for Cit H3 also detected NET-like structures as early as 1 h after histone stimulation (Supplementary Figure 3).

### Histone Induces NET Release *in vivo*

**Figure 4:**
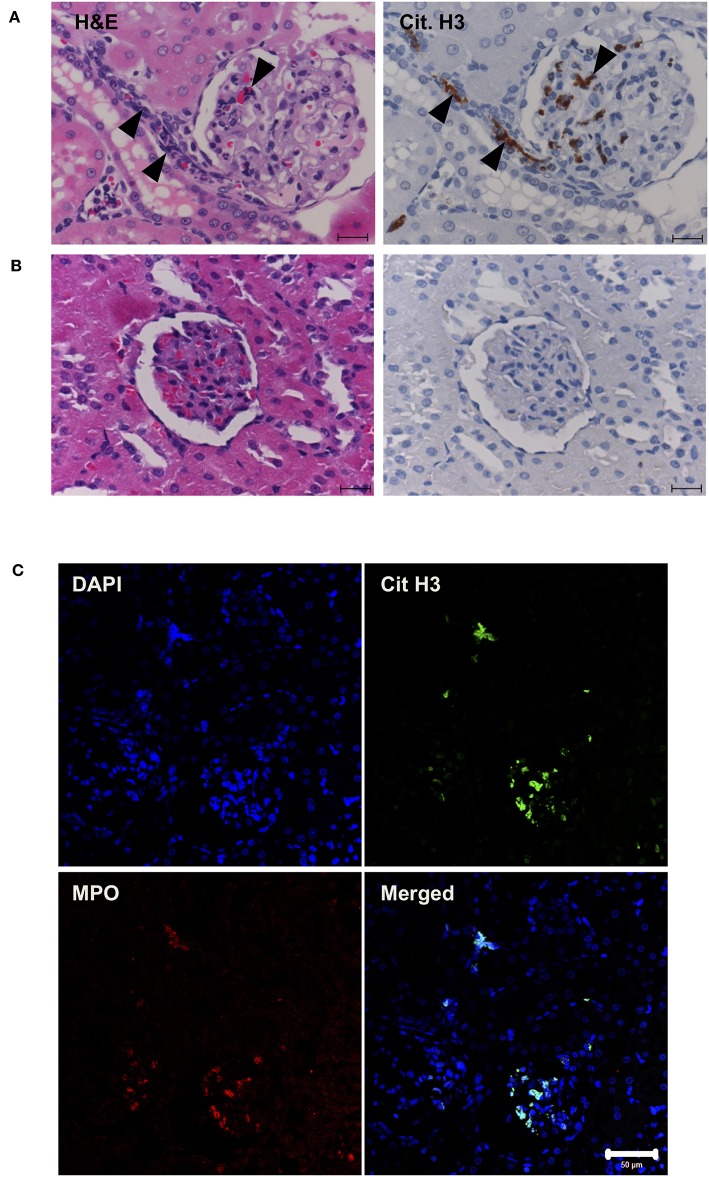
Histone induces NET release *in vivo*. Rats were infused with vehicle or 0.5 mg/kg/min histones for 4 h. Kidney sections of histone-infused rats showed nuclear smears in H&E staining and positive staining for Cit H3 in immunohistochemical staining **(A)**. Nuclear smears and Cit H3 positive staining were absent in vehicle-infused rats **(B)**. Scale bars, 20 μm. Kidney sections of histone-infused rats showed co-localization of Cit H3 and MPO **(C)**, suggesting the presence of NETs. Scale bar, 50 μm.

Next, *in vivo* experiments were carried out. Continuous intravenous infusion of 0.5 mg/kg/min histones into Sprague-Dawley rats resulted in serum histone H3 levels of 3.9 ± 1.6 μg/ml. Pathological analysis of kidney tissue sections at the end of histone infusion (4 h) showed accumulation of nuclear smears in capillaries (Figure 4A, arrowheads) which were absent in vehicle-treated rats (Figure 4B). Since a previous study suggested NETs could be detected as nuclear smears (36), we stained tissue sections for MPO and Cit H3. MPO and Cit H3 were found to be co-localized with collapsed DNA (Figure 4C), thus confirming the deposition of NETs in capillaries of histone-injected rats.

### rTM Suppresses Histone-Induced NET Release *in vitro* and *in vivo*

**Figure 5:**
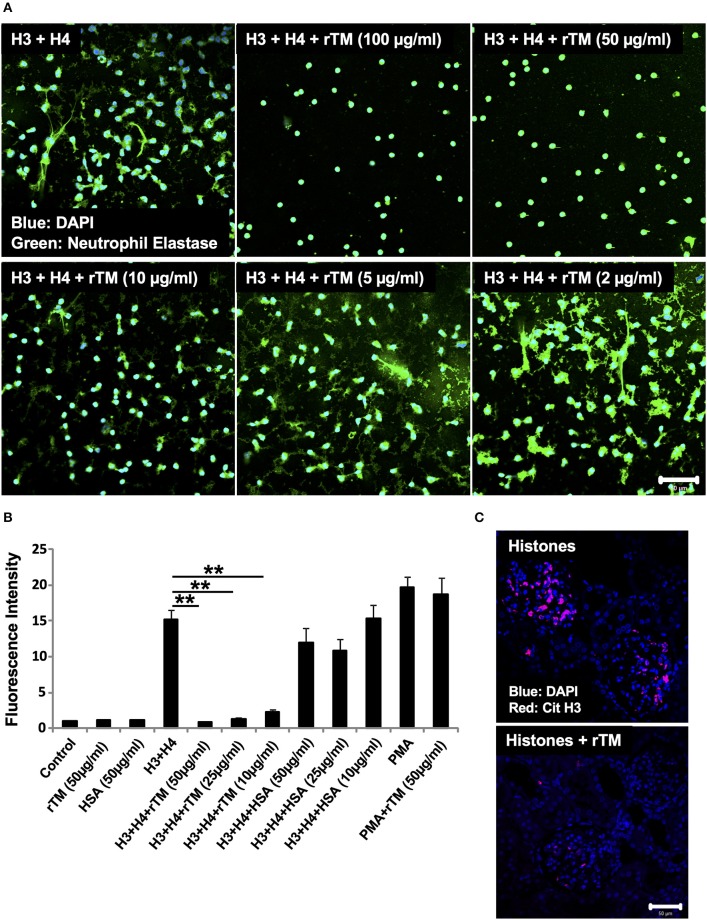
rTM suppresses histone-induced NET release *in vitro* and *in vivo*. Neutrophils isolated from healthy volunteers were treated with histones H3 and H4 (20 μg/ml) with or without rTM at the indicated concentrations **(A)**. The neutrophils were then incubated with a primary antibody against neutrophil elastase followed by an Alexa Fluor 488-conjugated secondary antibody. Nuclei were stained with DAPI. The presence of extracellular DNA was quantified by Sytox Green assays in the presence or absence of rTM at the indicated concentrations while human serum albumin at the same concentration was used as a negative control **(B)**. Kidney sections of histone-infused rats with or without rTM treatment were stained for Cit H3 **(C)**. Data are means ± SEM (*n* = 4). Scale bar, 50 μm. **p < 0.01.

In our previous study, rTM suppressed thromboembolism caused by extracellular histones (20). In the present study, we analyzed whether rTM can suppress histone-induced NET release. For this, we stimulated neutrophils with 20 μg/ml histones H3 + H4 for 4 h in the presence or absence of various concentrations of rTM (2–100 μg/ml). rTM suppressed histone-induced NET release in a dose-dependent manner with prominent suppression of NET release at 10 μg/ml or more (Figures 5A,B). rTM *per se* did not have an impact on neutrophil viability (Supplementary Video 1). Histone-induced NET release was also suppressed by rTM in our *in vivo* experiments. As shown in Figure 5C, histone-induced NET deposition as evidenced by Cit H3 staining in kidney sections was decreased in rTM-treated rats (group 4) compared with non-treated rats (group 3). As shown in Supplementary Figure 4, kidney dysfunction as evidenced by elevation of serum creatinine levels was observed 3 and 4 h after histone administration (group 3) but it was less pronounced in rTM-treated rats (group 4). Plasma tumor necrosis factor-α levels reached a peak at 2 h after histone administration and thus rTM treatment at 2 h did not influence cytokine levels in this model (data not shown).

### rTM Type 2 Suppresses Histone-Induced NET Release More Potently Than rTM

**Figure 6:**
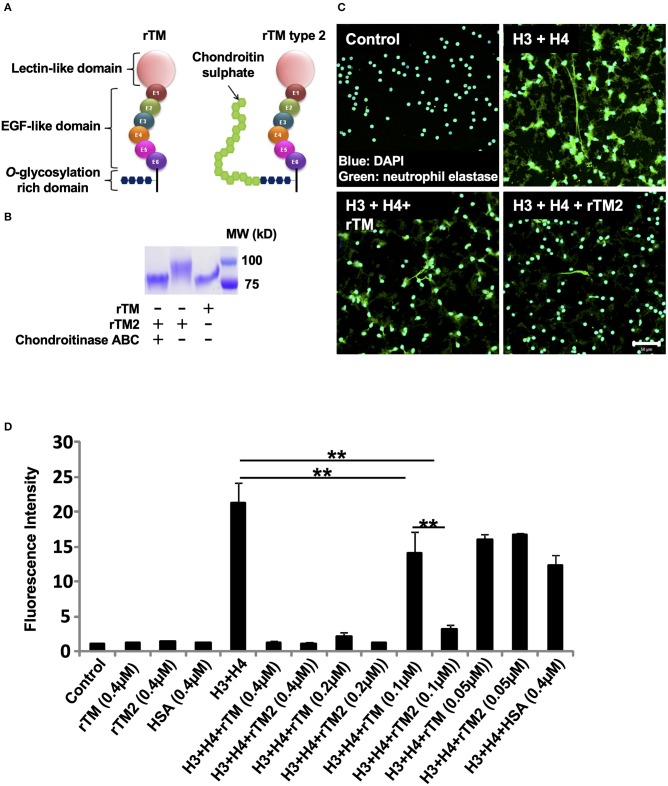
rTM2 suppresses histone-induced NET release more potently than rTM. rTM2 differs from rTM by the presence of a chondroitin sulfate side chain in the glycosylation site **(A)**. Incubation of rTM2 with chondroitinase ABC (100 μM) removes the chondroitin sulfate side chain **(B)**. Neutrophils isolated from healthy volunteers were left untreated or treated with histones H3 and H4 (20 μg/ml) with or without rTM or rTM2 at 0.1 μM **(C)**. The neutrophils were then incubated with a primary antibody against neutrophil elastase followed by an Alexa Flour 488-conjugated secondary antibody. Nuclei were stained with DAPI. The presence of extracellular DNA was quantified by Sytox Green assays in the presence or absence of rTM or rTM2 at the indicated concentrations while human serum albumin at the same concentration was used as a negative control **(D)**. Images and graphs are representative of three replicate experiments. Data are means ± SEM (*n* = 4). Scale bar, 50 μm. **p < 0.01.

We then examined another form of rTM, named rTM type 2 (rTM2), which has a chondroitin sulfate side chain attached to the *O*-glycosylation rich domain (Figure 6A). Treatment of rTM2 with chondroitinase ABC, which cleaves the chondroitin sulfate side chain, decreased the molecular weight of rTM2 to be similar to that of rTM (Figure 6B). Treatment with chondroitinase ABC had no effect on the molecular weight of rTM (data not shown). Immunofluorescence staining showed that rTM2 suppressed histone-induced NET release more potently than rTM (Figure 6C). In quantification experiments, we found that rTM2 suppressed histone-induced NET release more potently than rTM (Figure 6D). Potent inhibitory effects of rTM2 were also observed in histone-induced platelet aggregation (Supplementary Figure 5).

### Binding Assays With the QCM Twin Sensor System

**Figure 7:**
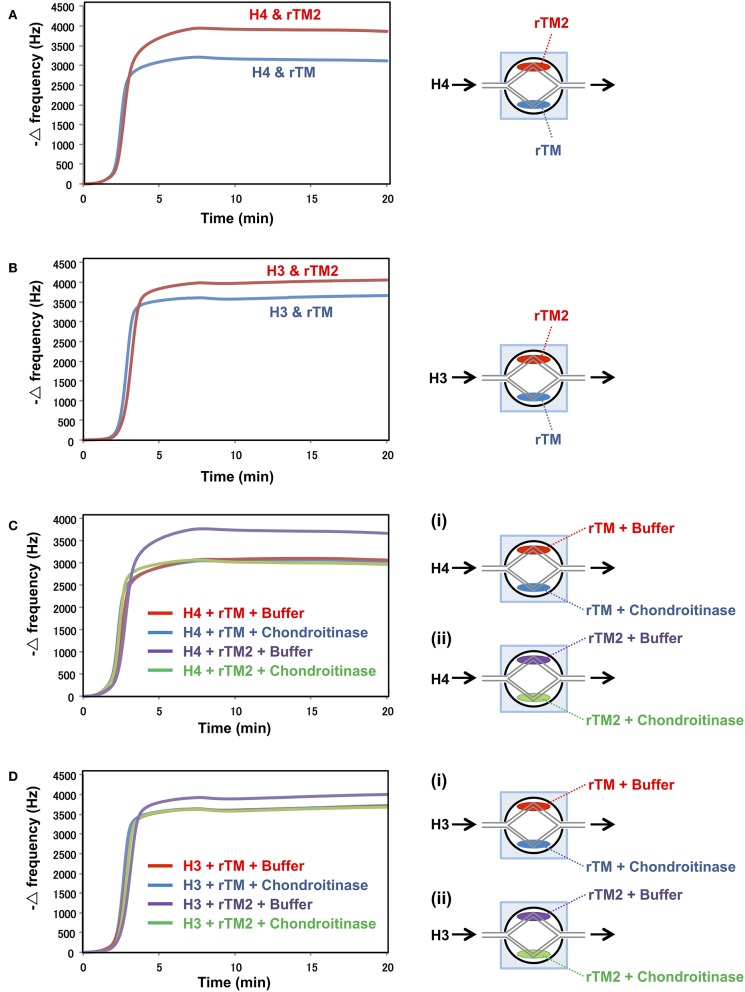
Binding assays with QCM twin sensor system. The two channels on a sensor chip were coated with either rTM2 (20 μM) or rTM (20 μM). The sensor chip was placed in NAPiCOS Auto system and perfused with histone H4 (50 μM) **(A)** or histone H3 (50 μM) **(B)**. The two channels on a sensor chip were coated with rTM in the presence or absence of chondroitinase ABC (Ci) or coated with rTM2 in the presence or absence of chondroitinase ABC (Cii). The sensor chip was placed in the NAPiCOS Auto system and perfused with histone H4 (50 μM) **(C)** or histone H3 (50 μM) **(D)**. The interactions between molecules were recognized as changes in frequency of a quartz crystal resonator. Representative data of two independent experiments are shown.

In a previous study, rTM was shown to bind to histones (20). Therefore, we compared the binding strengths of rTM and rTM2 to histones using the QCM twin sensor system. The interactions between molecules were recognized as changes in frequency of a quartz crystal resonator. As shown in Figures 7A,B, more prominent changes in frequency were observed during the interactions of histones with rTM2 compared with rTM, indicating that rTM2 bound to histones more strongly than rTM. Then, we removed the chondroitin sulfate side chain from rTM2 by incubation with chondroitinase ABC and compared the binding of rTM and rTM2 to histones. When the chondroitin sulfate side chain was removed by incubation with chondroitinase ABC, the binding of rTM2 to histones was similar to that of rTM (Figures 7C,D). These results suggest that the chondroitin sulfate side chain played an important role in the stronger binding of rTM2 with histones H3 and H4.

## Discussion

Histones are an integral part of the nucleus. Histone release during sepsis causes damage to endothelial cells and vascular injury (19, 21, 37). In a previous study, we showed that extracellular histones caused thromboembolism in mice, which was prevented by injection of rTM (20). NETs were associated with thrombosis in recent studies (6, 8, 10). Therefore, we analyzed whether histones can stimulate neutrophils to induce NET formation and whether rTM can prevent histone-induced NET formation.

Histones induced NET release in a dose-dependent manner. NET release was prominent at the 20 μg/ml concentration of histones i.e., 10 μg/ml for each of histones H3 and H4. It was reported that the plasma concentration of histone H3 during sepsis can reach about 15 μg/ml (21). Therefore, the concentrations used in our experiments might be in accordance with the physiological concentrations. NET formation was observed from 5 μg/ml histone H3 and histone H4 individual concentrations, also supporting the concentrations of histones used in our *in vitro* experiments. However, we used the 10 μg/ml concentration of histones for most of our experiments to ensure that the production of NETs was easily observed. Furthermore, as shown in Figure 1, we found that although NETs were visible by immunostaining at the total histone concentration of 10 μg/ml, extracellular DNA was only measurable in quantification assays at the concentration of 20 μg/ml. The lower sensitivity of DNA dye in the presence of extracellular histones may explain the discrepancy.

We also found that histone-induced NET release was NADPH oxidase-independent, because it was not suppressed by pre-treatment with NADPH oxidase inhibitor DPI, while DPI inhibited PMA-induced NET release almost completely. PMA-induced NET release is dependent on NADPH oxidase, although the requirement of NADPH oxidase differs depending on the stimulus received (33, 34). Stimuli like calcium ionophores and monosodium urate crystals induce NET formation that is NADPH-independent. Histones may follow similar mechanism to that of calcium ionophores or monosodium urate crystals for NET release. Since the NET release mechanism for histones differs from that for PMA, we measured the kinetics of histone-induced NET release. PMA at a high concentration (200 nM) released NETs at 3 h while PMA at a low concentration (50 nM) induced NET release at about 4 h after stimulation. However, histones induced NET release as early as 1 h after stimulation. This result is similar to that for NET release induced by monosodium urate crystals, which generated NETs earlier than PMA (33). In one experiment, we found that micrococcal nuclease (MNase) treatment dissociated NET structures in the case of PMA-induced NET release, but did not dissociate NET structures in the case of histone-induced NET release, showing that the NET structures released by histone treatment differed from those released by PMA treatment (data not shown).

Histones were reported to act through TLR2 and TLR4 during the death of epithelial and endothelial cells during inflammation (15, 18). However, we could not show whether histone-induced NET release was TLR-dependent in this study. In our *in vitro* experiments, NET production after treatment with anti-TLR2 and anti-TLR4 antibodies was similar to that after treatment with control IgG (data not shown). Since histones bind easily with immunoglobulins (38, 39), binding of histones with the antibodies leading to insufficient available histones to induce NET production. As neutrophils are very short-lived cells, it was very difficult to knockdown TLRs and perform such experiments. However, it could be possible to confirm whether histones cause NET release by neutrophils through TLRs using TLR knockout mice in future studies.

Histone-induced NET release was also evaluated in *in vivo* experiments. We confirmed the presence of NETs in various tissues of rats infused with histones by staining for Cit H3. Cit H3-positive staining was absent in the liver and spleen but present in the lung and kidney. Cit H3-positive staining was decreased in the kidney tissues of rats treated with rTM and histones compared with rats treated with histones only. In the lungs, Cit H3 was present in both groups in similar amounts (data not shown).

Thrombomodulin is considered to be a part-time proteoglycan, which may exist with or without a glycosaminoglycan (GAG) chain. The presence of a chondroitin sulfate side chain may confer profound anticoagulant properties (40). In this context, rTM does not have GAG while rTM2 has a chondroitin sulfate side chain attached to the *O*-glycosylation rich domain. We compared the strengths of these two forms for suppression of histone-induced NET release, and found that histone-induced NET release was suppressed by rTM2 more efficiently. Similarly, histone-induced platelet aggregation was suppressed more strongly by rTM2. Since the difference between rTM and rTM2 is the presence of a chondroitin sulfate side chain and histones were reported to bind to GAGs (41), the presence of extra GAG may have led to stronger binding of rTM2 to histones. In fact, binding assays using NAPiCOS Auto system showed that histones H3 and H4 both bound strongly to rTM2 and removal of the GAG chain on rTM2 decreased the histone binding to a similar level to the binding to rTM. These findings suggest that the presence of GAG may be one reason for the stronger binding of rTM2 with histones and thus the more potent ability of rTM2 to suppress histone-induced NET release. However, rTM, which does not have GAG, still binds to histones, suggesting that other domains of thrombomodulin may also contribute to the binding to histones (Supplementary Figure 6).

Our study has revealed the important roles of histones for induction of NET formation *in vitro* and *in vivo*. These findings may be important because sepsis is associated with high plasma histone levels (21) and is also complicated by thrombosis (42). Histones are well-known mediators of endothelial cell death during sepsis, but may also have an important role in thrombosis development (8, 43, 44). Use of rTM for DIC is already approved in Japan. Inhibition of histone-induced NET release may be one mechanism for the efficacy of rTM in the treatment of sepsis.

## Data Availability Statement

All datasets generated for this study are included in the manuscript/Supplementary Files.

## Ethics Statement

The studies involving human participants were reviewed and approved by Ethics Committee of Kagoshima University. The patients/participants provided their written informed consent to participate in this study. The animal study was reviewed and approved by Shin Nippon Biomedical Laboratories, Kagoshima, Japan.

## Author Contributions

BS performed research, analyzed data, generated the figures, and wrote the manuscript. TI designed experiments and wrote the manuscript. MK, TT, and TN contributed *in vitro* studies. MY contributed in binding assays with QCM twin sensor system. IM designed and organized the experiments and approved the final manuscript.

### Conflict of Interest

TI and IM hold endowed faculty positions on thrombosis research and have received funds from Asahi Kasei Pharma, a pharmaceutical company manufacturing recombinant thrombomodulin. The funder was not involved in the study design, collection, analysis, interpretation of data, the writing of this article or the decision to submit it for publication. There are no further patents, products in development or marketed products to declare. The remaining authors declare that the research was conducted in the absence of any commercial or financial relationships that could be construed as a potential conflict of interest.

## Supplementary Material

Supplementary Video 1 rTM *per se* does not have an impact on neutrophil viability while it suppresses histone-induced NETosis.
