# Supplementary material for: Recombinant Thrombomodulin Suppresses Histone-Induced Neutrophil Extracellular Trap Formation
Source: Front Immunol. 2019 Oct 29;10:2535. doi: 10.3389/fimmu.2019.02535 (PMC6828967; doi:10.3389/fimmu.2019.02535)
Supplement: Supplementary file 1 [file Data_Sheet_1.PDF]

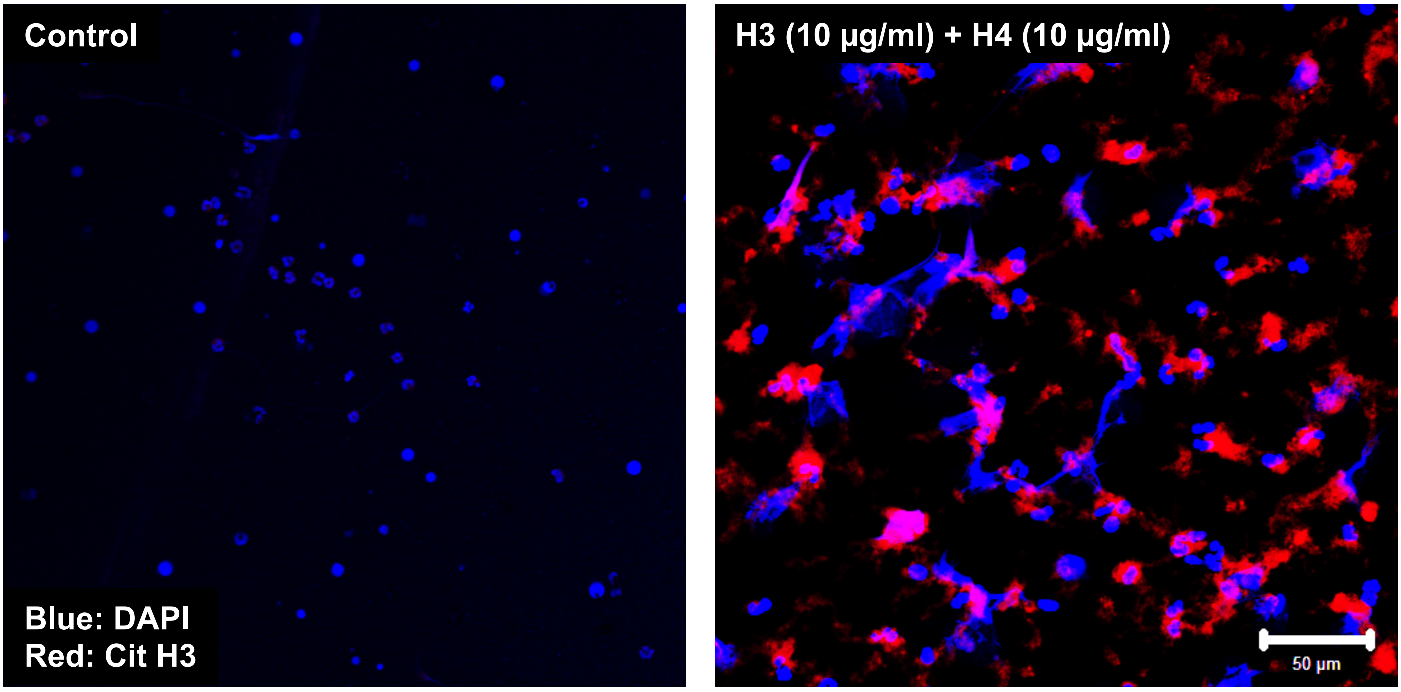

Supplementary Figure 1 | NET-like structures are positive for citrullinated histone H3 staining. Neutrophils were incubated in the absence or presence of histones H3/H4 for 4 h. The neutrophils were then incubated with a primary antibody against Cit H3 followed by an Alexa Flour 594-conjugated secondary antibody. Nuclei were stained with DAPI. Scale bar, 50 µm.

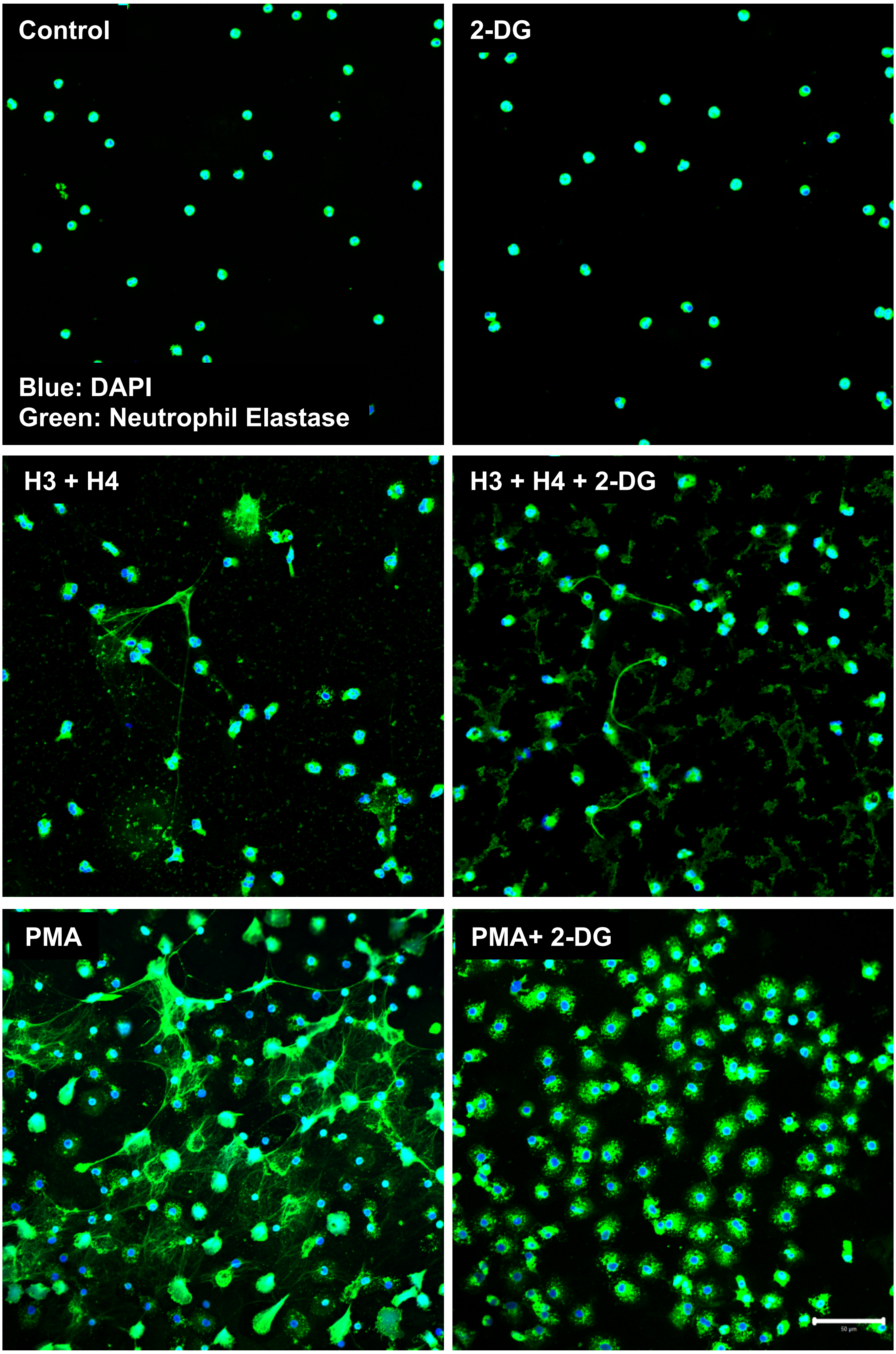

Supplementary Figure 2 | Pharmacological inhibition of glycolysis does not inhibit histone-induced NET. Isolated neutrophils were treated with PMA (200 nM) or histones H3 and H4 (20 µg/ml) in the absence or presence of 2-Deoxy Glucose (2-DG). The neutrophils were then incubated with a primary antibody against neutrophil elastase followed by an Alexa Flour 488-conjugated secondary antibody. Nuclei were stained with DAPI. Images are representative of three replicate experiments. Scale bar, 50 µm.

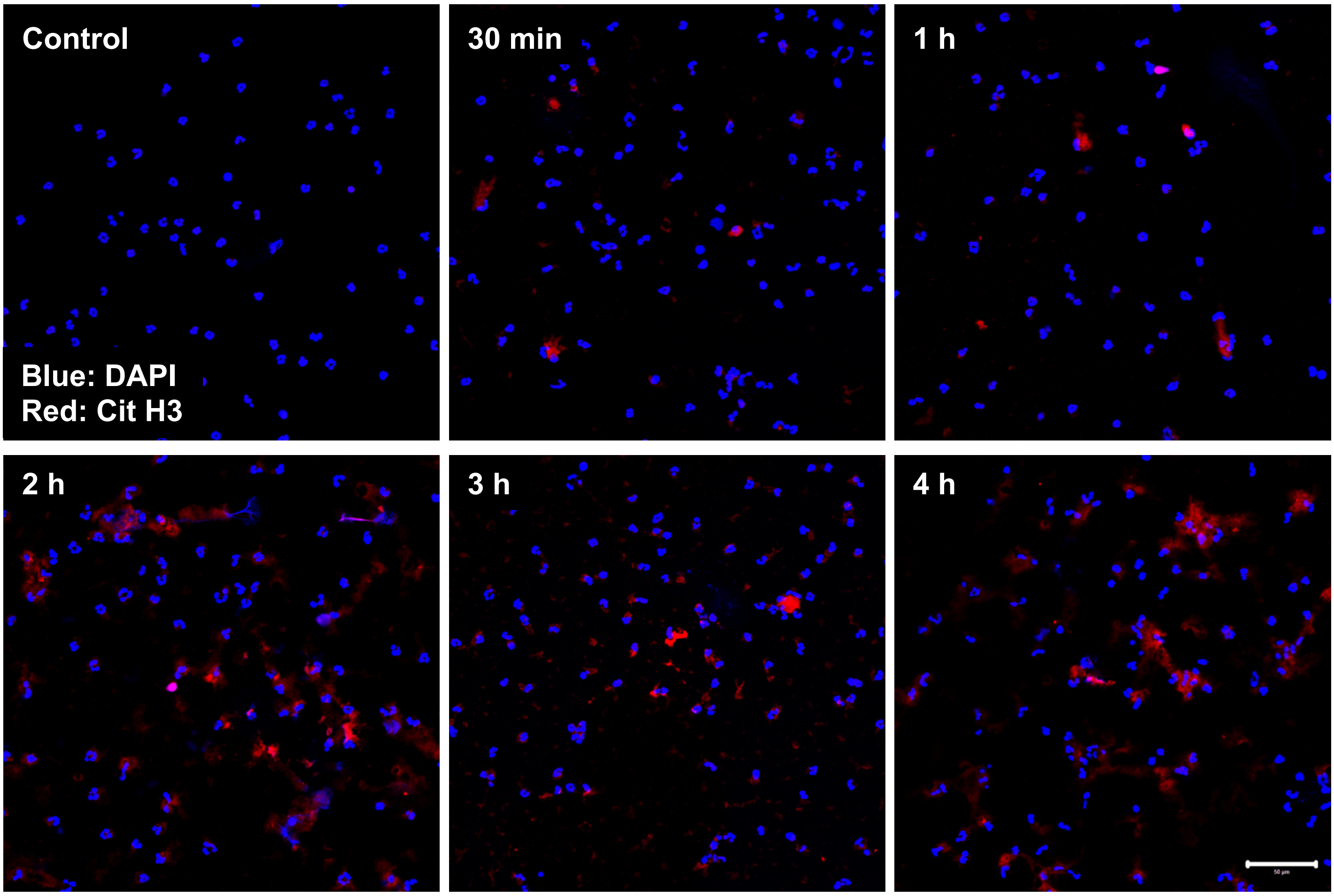

Supplementary Figure 3 | Histone induces NET release in neutrophils as early as 1 h after stimulation. Isolated neutrophils were left untreated or treated with histones H3 and H4 (20 μg/ml) for the indicated time intervals. The neutrophils were then incubated with primary antibody against Cit H3 followed by an Alexa Flour 594-conjugated secondary antibody. Nuclei were stained with DAPI. Images are representative of two replicate experiments. Scale bar, 50 μm.

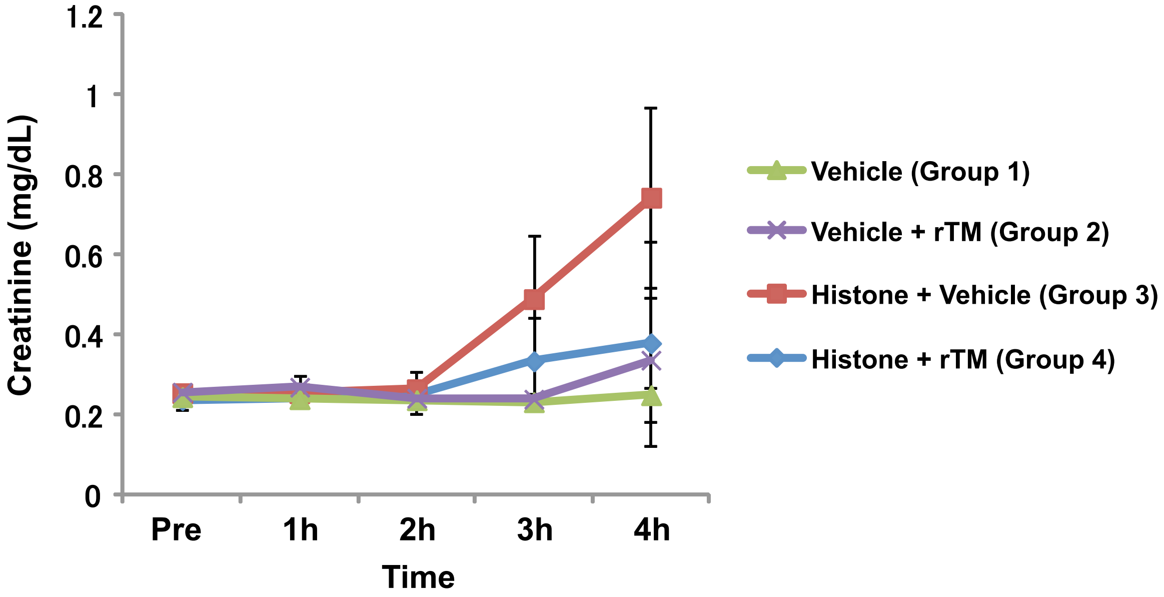

Supplementary Figure 4 | rTM alleviates renal dysfunction caused by histones infusion. Rats were infused with vehicle (groups 1 and 2) or 0.5 mg/kg/min histones (groups 3 and 4) for 4 h. Groups 2 and 4 were given a bolus injection of 1 mg/kg rTM at 2 h. Serum creatinine levels were measured at the indicated time intervals.

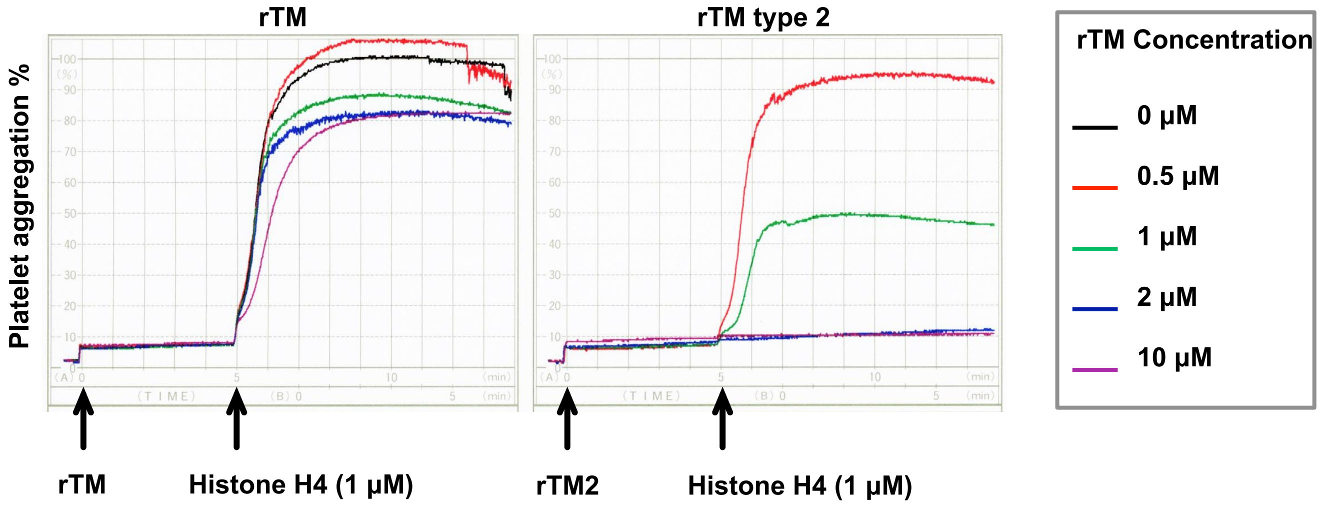

Supplementary Figure 5 | Inhibition of histone H4-mediated platelet aggregation by rTM and rTM2. Washed platelets were preincubated with rTM or rTM2 (0.5-10 µM) and then stimulated with 1 µM of histone H4**.** rTM2 inhibited histone-induced platelet aggregation more potently. Graphs are representative of two replicate experiments.

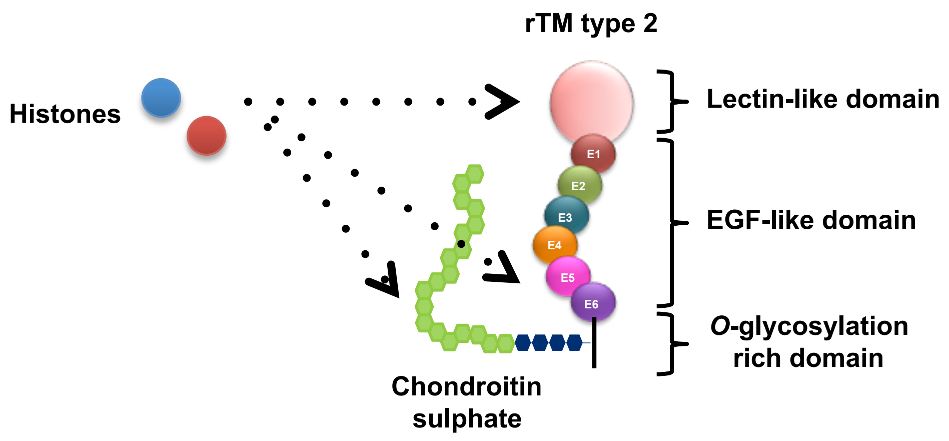

Supplementary Figure 6 | Possible histone-binding sites in rTM type 2. Chondroitin sulfate side chain plays an important role in the stronger binding of rTM type 2 with histones. However, rTM, which does not have chondroitin sulfate, still binds to histones, suggesting other domains may also contribute to the binding. The N-terminal lectin-like domain is a candidate because this domain is important in binding with DAMPs and LPS. The negatively charged fourth, fifth, and sixth EGF-like domains may also interact with positively charged histones.
